# The relationship between social support and physical and mental health in an older male population: Evidence from China Health and Retirement Longitudinal Study (CHARLS)

**DOI:** 10.1371/journal.pone.0330109

**Published:** 2025-08-27

**Authors:** Yanling Li, Yi Xiang

**Affiliations:** 1 School of Public Administration and Law, Hunan Agricultural University, Changsha, China; 2 School of Public Policy and Administration, Xi ‘an Jiaotong University, Xi ‘an, China; Thammasat University, THAILAND

## Abstract

In the context of increasing population aging and decreasing birth rate, it is of great practical significance to explore the impact of social support on the health of elderly men, which is of great practical significance to smoothly promote the strategy of healthy China and actively implement the strategy of population aging. Using the newly released 2020 China Health and Retirement Longitudinal Study (CHARLS) data for a total of 1,510 study participants, this paper analyzes the impact of social support on the health of elderly men using the Oder Probit, OLogit, and PSM models, and then explores the variability across age stages and literacy levels, and eliminates the propensity score matching modeling by endogeneity problems caused by sample selectivity bias. The study shows that social support significantly improves the health of older men in the context of a deepening aging process, showing positive improvements in both self-assessed health and mental health. The robustness test (by replacing the econometric model) further confirmed the reliability of the findings. Heterogeneity tests, on the other hand, revealed significant differences in the impact of social support on the health of male older adults: its health-enhancing effect was more pronounced in less literate male older adults compared to the more literate group. The results of the endogeneity analysis showed that the PSM model effectively mitigated the endogeneity problem of the model. Failure to deal with endogeneity would lead to underestimation of two aspects: first, the health-enhancing effect of social support on self-assessed health of male older adults; and second, its improvement effect on mental health. Therefore, the Chinese government should pay more attention to the key role of social support in improving the health of male older adults, and take diversified and innovative initiatives to focus on enhancing social support in order to more effectively promote the goal of healthy aging.

## 1. Introduction

With the acceleration of global population aging, the health issue of the elderly has become a hot topic widely concerned by the international community and academia [1–3]. In China, as the country with the largest population in the world, the aging trend is particularly significant. The rapid growth of the elderly population not only has a profound impact on the social and economic structure, but also poses new challenges to the health protection of the elderly [4–5]. At the same time, rising childcare costs and changes in Chinese society’s culture and values have led to record low marriage and fertility rates, and the number of elderly people living alone has increased over the years [6–7]. In this context, it is particularly important to explore how to effectively promote the health of the elderly, especially for groups with specific cultural and social backgrounds, such as Chinese elderly men. According to data released by the National Bureau of Statistics of China, by the end of 2023, the elderly population aged 60 and above accounted for 21.1%, and the elderly population aged 65 and above accounted for 15.4%. With the further increase of the proportion of the elderly population, the degree of aging continues to expand, and the health status of the elderly is worrying [8–9]. According to the latest statistics from China’s National Health Commission, about 190 million elderly people in China suffer from chronic diseases, with 75 percent of those aged 60 and above suffering from at least one chronic disease and 43 percent suffering from two or more diseases at the same time. Chronic diseases are defined as diseases that are long-lasting, slow to develop, usually not fully curable and require long-term management. They are a major threat to human health and the leading cause of death and disability worldwide (accounting for more than 70% of global deaths). Unlike acute diseases (e.g., influenza, bone fractures), chronic diseases are usually progressive and symptoms may last for months, years or even a lifetime, e.g., diabetes mellitus, hypertension, malignant tumors. The above data show that the health status of the elderly is not optimistic.

To this end, the Chinese government has continuously introduced several policy measures to improve the health of the elderly. As early as 2016, the Chinese government put forward the Healthy China Strategy, elevating population health to an important level of national strategy [10]. In 2022, the report of the 20th National Congress of the Chinese Government once again emphasized that it is necessary to promote the construction of a healthy China, implement the national strategy of actively responding to the aging population, develop the elderly care service and the elderly care industry, optimize the service for the elderly, and promote the realization of basic elderly care services for all the elderly. As an important group of the elderly, Chinese elderly men play an important role in the social structure. They are not only an important pillar of the family, but also an important participant in social development [11]. However, with the growth of age, elderly men are faced with multiple challenges such as physiological function decline, psychological status change and social role change, which often have a negative impact on their health [12]. As an external resource, social support can relieve the life pressure of the elderly and enhance their coping ability by providing emotional comfort, material help and information guidance, thus exerting a positive impact on their health [13]. Therefore, in-depth research on the impact of social support on the health of elderly Chinese men not only helps to reveal the inherent law of elderly health but also provides an important basis for formulating more scientific and reasonable elderly health policies. This is of great practical significance for the Chinese government to actively implement the strategy of population aging, comprehensively promote the strategic construction of healthy China, and formulate the population policy in the new era.

At present, some studies have explored the relationship between social support and population health [14–16]. If there are studies to analyze the impact of social support on the health promotion behaviors of the elderly in different ages in Shanghai, it is found that social support has a significant impact on the health of the elderly in different ages [17]. Some studies have also analyzed the impact of social support on the mental health of poor vocational students, and found that social support is an important factor affecting the mental health of poor vocational students [18]. However, a survey of existing studies shows that, on the one hand, there are few studies on the special group of elderly men; On the other hand, there is a lack of heterogeneity analysis of the relationship between social support and population health from the perspectives of age and educational level. Understanding the relationship between social support and the health of older men is particularly important in the context of deepening aging in our country and record low marriage and fertility rates. Based on the above background, this paper focuses on the following core questions: does social support, as a key social resource, significantly influence the health status of male older adults in the context of China’s deepening population aging? Is the effect heterogeneous among male older adults with different literacy levels? Using data from the 2020 China Health and Retirement Longitudinal Study (CHARLS), this paper empirically examines the effect of social support on the health of male older adults, and ensures the reliability of the findings through robustness tests, heterogeneity analyses, and propensity score matching (PSM) modeling (used to correct for the endogeneity problem caused by sample selectivity bias). This study not only helps to analyze the internal mechanism of social support on men’s health, but also provides solid empirical evidence and policy references for the promotion of healthy aging and active aging strategies in China.

## 2. Materials and methods

### 2.1. Data sources

The data in this paper are from the newly published 2020 China Health and Retirement Longitudinal Study (CHARLS). This data is a public database, all users can in the Centre for Social Research and Studies of Peking University’s official website (https://charls.charlsdata.com/pages/data) to apply for and obtain. The database aims to collect a set of high-quality micro-data representing households and individuals aged 45 and above in China to analyze the problem of population aging in China and promote interdisciplinary research on aging. The CHARLS national baseline survey was conducted in 2011, covering 150 county-level units, 450 village-level units, and 17,000 people in about 10,000 households. This study was approved by the Ethics Committee of Peking University (IRB approval number IRB00001052–11015). Written informed consent to participate in this study was provided by the participants or the participant’s legal guardian/next of kin. The 2020 questionnaire included information on the respondents’ demographics, family status, economic base, health and personal life characteristics. According to the subject and content of the study, this paper selects elderly men (age ≥ 60 years) as analysis samples and then optimizes the samples according to the selection of control variables, eliminating the missing values, outliers and invalid variables of the samples, and obtaining 1501 valid samples.

### 2.2. Variable design

The dependent variable. The health status of male older adults was used as the dependent variable in this study. Academics use multiple criteria to measure individual health, among which Self-rated health (SRH) has been commonly adopted because its validity has been confirmed by extensive research [19–21]. However, along with the development of society, the importance of mental health has become increasingly prominent. Therefore, taking into account the existing literature and the main purpose of this study, this paper adopts the two dimensions of self-rated health and mental health together to measure the health status of male older adults. The data were obtained from the 2020 China Health and Retirement Longitudinal Study (CHARLS). Self-assessed health: According to the question DA001, how do you think your health is? (options: very good, good, fair, bad, very bad), assigned a value of 1 (very good) to 5 (very bad), with higher scores indicating poorer self-assessed health. Mental Health: Based on question DC018. (Options: rarely or not at all, not too much, sometimes or half the time, most of the time), assigned a value of 1 (rarely or not at all) to 4 (most of the time), with the following meanings: 1 = good mental health (rarely depressed); 2 = fair mental health (rarely depressed); 3 = poor mental health (sometimes depressed); 4 = poor mental health (often depressed); 4 = poor mental health (often depressed). health status is poor (often depressed), i.e., the higher the score, the poorer the mental health status.

The independent variable. Social support is the core independent variable of this study. As a multidimensional and complex concept, social support covers multiple forms of help from different social networks (e.g., family, friends, neighbors, community organizations, etc.). Based on the nature of the source of support, it can be categorized into formal social support and informal social support [22]. Among them, formal social support refers to the institutionalized and professionalized support provided by formal organizations such as government, charitable organizations, social work agencies, and units [23], which is stable and reliable; while informal social support originates from interpersonal mutual support networks such as family, friends, neighbors, and co-workers [24], which is characterized by spontaneity, emotionality, and flexibility. Based on established research, this paper measures social support from both formal and informal dimensions. This was done as follows: using data from the 2020 China Health and Retirement Longitudinal Study (CHARLS), formal social support was measured by the question “Have you ever received home healthcare services?” (acceptance = 1, no acceptance = 0); informal social support was measured by the question “Do respondents receive financial support from their children?” (support = 1, no support = 0), following the conventions of the literature.

Control variables. The theoretical model of health ecology shows that the health of a population is not only affected by social factors, but also closely related to its own personal characteristics and lifestyle [25–27]. Social support, as the core independent variable, acts primarily at the interpersonal level. However, individual-level characteristics (personal traits and lifestyle) are not only direct influences on health, but may also be associated with social support (e.g., a healthy lifestyle may influence social participation), thus potentially confounding the causal relationship between social support and health outcomes. To more accurately estimate the independent effect (net effect) of social support on physical and mental health among older men, this paper includes gender, age, place of residence, literacy, marital status, health care participation, smoking status, and alcohol consumption as control variables. For example, in terms of age, which is an important determinant of health, the decline in physiological function, increased risk of chronic disease, and significant changes in social roles with age within the elderly population directly affect physical and mental health. In terms of place of residence, urban-rural differences may be reflected in the accessibility of healthcare resources, community support services, patterns of social interaction, environmental quality, and lifestyle, all of which may independently affect health levels. The inclusion of these variables in the model in this study aims to control as comprehensively as possible for important health influences known at the individual level and to reduce estimation bias due to the omission of important confounding variables. The definitions and statistics of specific variables are shown in Table 1.

**Table 1:** Descriptive statistics of variables.

Variable	Definition	N	Mean	SE	Max	Min
**Dependent variable**						
Self-rated health	Very good = 1, good = 2, fair = 3, bad = 4,5 = very bad	1501	2.712	1.009	5	1
Mental health	1 = good, 2 = fair, 3 = bad, 4 = very bad	1501	1.656	0.955	4	1
**Independent variable**						
Formal social support	No = 0, yes = 1	1501	0.171	0.376	1	0
Informal social support	No = 0, yes = 1	1501	0.967	0.178	1	0
**Control variable**						
Age	Unit: year	1501	65.034	0.626	77	60
Place of Residence	Home = 1, nursing home = 2, hospital = 3	1501	1.129	0.607	4	1
Education level	Illiteracy = 1, primary = 2, secondary = 3, college and above=4	1501	2.085	0.354	4	1
Marital status	Married = 1, divorced = 2, widowed = 3, unmarried = 4	1501	1.157	0.555	4	1
Medical insurance	No = 0, yes = 1	1501	0.987	0.115	1	0
Smoking	No = 0, yes = 1	1501	0.134	0.341	1	0
Drinking	No = 0, yes = 1	1501	0.545	0.498	1	0

### 2.3. Model design

The dependent variable of this paper is the health status of elderly men. Since the measurements of self-rated health and mental health are classified variables, this paper used Ordered Probit regression model [28] for analysis. In this paper, the Ordered Probit regression model is set as follows:


$${\text{Health}}_{\text{i}}^{\text{*}}=\alpha +\beta {\text{social support}}_{\text{i}}+\gamma {\text{Z}}_{\text{i}}\text{+}\varepsilon_{\text{i}}$$ (1)

In formula (1), ${\text{Health}}_{\text{i}}^{\text{*}}$ is the health status of elderly men, ${\text{social support}}_{\text{i}}$ is the degree of social support, ${\text{Z}}_{\text{i}}$ is the control variable affecting the health status of elderly men, and $\varepsilon_{\text{i}}$ is the random disturbance term. β is the regression coefficient, that is, the effect of social support on the health of elderly men.

The behavior of male older adults in receiving social support may be affected by factors such as gender, age, literacy, and marital status, leading to sample selectivity bias, which in turn affects the model estimation results. In order to effectively mitigate the resulting estimation bias, this paper adopts the Propensity Score Matching (PSM) model [29] to estimate the net effect of social support on the health of male older adults. The model maximizes the control of the effect of observable confounders by dividing the sample into treatment (receiving support) and control (not receiving support) groups and matching based on propensity scores. The model Settings are as follows:


$${\text{Y}}_{\text{i}}\text{=}{\text{Y}}_{\text{0i}}\text{+(}{\text{Y}}_{\text{1i}}\text{-}{\text{Y}}_{\text{0i}}\text{)}{\text{D}}_{\text{i}}$$ (2)

$$\text{ATT=E(}{\text{Y}}_{\text{1i}}\text{-}{\text{Y}}_{\text{0i}}|{\text{D}}_{\text{i}}\text{=1)}$$ (3)

In formula (2), ${\text{D}}_{\text{i}}$ is the processing variable. When the value is 1, it means that individual i is in the experimental group; when the value is 0, it means that individual i is in the processing group. The core explanatory variables of this paper are two groups, in which the treatment group is the elderly male with social support, and the control group is the elderly male without social support. Formula (3) represents the average treatment effect of the treatment group, that is, the pure effect of social support on the health of elderly men.

## 3. Results

### 3.1. Descriptive statistics

As shown in the results of descriptive statistics in Table 1, the average age of the interviewed male elderly was 65.034 years old, and their ages were mainly concentrated in the 60–77 age range. In terms of health status, the average self-assessed health score was 2.712, and the average mental health score was 1.656 (the higher the score, the worse the health status), which indicates that the self-assessed health of the sample was in the middle to lower level, while the frequency of mental health problems was relatively low. In terms of demographic and social characteristics: there were significant differences in the composition of educational attainment, with elementary school education predominating (65.65%), followed by secondary school (20.07%) and illiteracy (10.73%), and the lowest proportion of respondents with university education and above (3.55%); the vast majority of respondents lived at home (80.84%), with 10.13% and 9.03% living in nursing homes and hospitals, respectively; participation in health insurance is high, with 85.87% of those insured and 14.13% of those not insured. In terms of lifestyle: the majority of respondents have smoking habits (86.61%), while only 13.39% are non-smokers; the proportion of those who drink alcohol (54.57%) and those who don’t (45.43%) are more or less the same. Overall, the sample was characterized by a middle-aged and elderly male group that was predominantly home-bound, insured, with elementary school education, and generally with a smoking habit.

### 3.2. Results of baseline regression

Table 2 reports the results of the baseline regression on the impact of social support on the health of older men. Specifically: model (1) (core independent variable only) shows that the coefficient of formal social support on self-rated health is −0.641 (significant at the 1% level) and the coefficient of informal social support is −0.282 (significant at the 5% level), suggesting that both types of social support significantly improve self-rated health among older men (i.e., the group that has the support has better self-rated health). Model (2) After incorporating control variables such as age, place of residence, and literacy based on model (1), the coefficient of formal social support basically stabilizes at −0.642 (significant at the 1% level), and the coefficient of informal social support slightly decreases to −0.219 (still remains significant at the 5% level), which further confirms the positive effect of social support on self-assessed health. Model (3) examines the effect of social support on mental health and shows a coefficient of −0.292 (significant at the 1% level) for formal social support and a coefficient of −0.189 (significant at the 10% level) for informal social support, confirming that social support likewise has a significant contributory effect on mental health. Model (4) After adding control variables to model (3), the coefficient of formal social support decreased slightly to −0.291 (significant at the 1% level), and the coefficient of informal social support decreased to −0.133 (significant at the 10% level), again verifying that social support has a robust effect on the improvement of mental health. In summary, the baseline regression results consistently showed that both formal and informal social support significantly improved self-rated health and mental health among male older adults, with or without controlling for individual characteristics (a significant negative coefficient means lower health scores, i.e., better health), and that the role of formal social support was generally stronger and more statistically significant.

**Table 2:** Results of baseline regression.

Variable	Model (1)	Model (2)	Model (3)	Model (4)
	Self-rated health	Self-rated health	Mental health	Mental health
Formal social support	−0.641^***^ (0.074)	−0.642^***^ (0.075)	−0.292^***^ (0.079)	−0.291^***^ (0.079)
Informal social support	−0.282^**^ (0.156)	−0.219^**^ (0.164)	−0.189^*^ (0.181)	−0.133^*^ (0.190)
Age		−0.003^*^ (0.046)		0.027^*^ (0.047)
Place of Residence		−0.034 (0.046)		0.020 (0.050)
Education level		−0.136^*^ (0.079)		−0.071^*^ (0.089)
Marital status		0.050^*^ (0.050)		0.034^*^ (0.056)
Medical insurance		−0.312 (0.256)		0.312 (0.317)
Smoking		0.089 (0.083)		0.087^*^ (0.092)
Drinking		−0.274^**^ (0.056)		−0.105^*^ (0.062)
N	1501	1501	1501	1501
Adj-R^2^	0.0194	0.0272	0.0046	0.0065

Note:

*,

**and

***indicate significance at 10%, 5% and 1%, respectively.

### 3.3. Robustness test

Preliminary results from the baseline tests indicated validation of the effect of social support on the health of male older adults. However, to rule out potential endogenous confounding (e.g., omitted variables or heterogeneous relationships), this study conducted robustness tests. Specifically, we reassessed the effect of social support on the health of male older adults by replacing the OLogit model (using the OLS model). The test results confirmed the robustness of the regression model (see Table 3 for specific estimation results). The results of the robustness test (using the alternative OLogit model) further validated the impact effect of social support: in the self-rated health dimension, formal social support showed a significant negative effect on male older adults (p < 0.01), and informal social support also showed a negative effect (p < 0.10); in the mental health dimension, formal social support had a significant inhibitory effect on rural older adults (p < 0.01), and the negative effect of informal social support on male older adults also passed the significance test (p < 0.05). These findings are highly consistent with the results of the baseline regression and together confirm the robustness of the impact of social support on the health of the older population.

**Table 3:** Robustness test results.

Variable	Model (5)	Model (6)	Model (7)	Model (8)
	Self-rated health	Self-rated health	Mental health	Mental health
Formal social support	−1.123^***^ (0.134)	−1.126^***^ (0.135)	−0.481^***^ (0.132)	−0.479^***^ (0.132)
Informal social support	−0.482^*^ (0.274)	−0.334^*^ (0.287)	−0.317^**^ (0.310)	−0.228^**^ (0.320)
Age		0.003^*^ (0.074)		0.059^*^ (0.074)
Place of Residence		−0.047 (0.077)		−0.038 (0.082)
Education level		−0.253^*^ (0.134)		−0.079^*^ (0.145)
Marital status		0.079^*^ (0.090)		0.014^*^ (0.097)
Medical insurance		−0.573 (0.429)		0.690 (0.588)
Smoking		0.174 (0.148)		0.108^*^ (0.155)
Drinking		−0.467^**^ (0.099)		−0.138 (0.105)
N	1501	1501	1501	1501
Adj-R2	0.0185	0.0261	0.0045	0.0059

Note:

*,

**and

***indicate significance at 10%, 5% and 1%, respectively.

### 3.4. Heterogeneity analysis

The influence of social support on the health of older men with different education levels may be quite different. Therefore, this paper analyzes the heterogeneity of the health of elderly men from the perspective of education level differences. The analysis of heterogeneity in education level showed that there were significant group differences in the impact of social support on the health of male older adults. Based on the characteristics of the sample distribution and the real-world context, this study divided the education level into two categories: elementary school and below, and secondary school and above. The results in Table 4 show that at the level of formal social support, it has a significant contribution to the health of male older adults at the primary and below education level (p < 0.05), but not at the secondary school and above group; informal social support shows the same pattern (p < 0.05 in the elementary school and below group, and the secondary school and above group did not pass the test of significance). This suggests that there is a clear educational gradient in the health effects of social support (whether formal or informal): older men with lower levels of education benefit more significantly from social support.

**Table 4:** Heterogeneity test at different educational levels.

Variable	Primary and below		Above primary school	
	Self-rated health	Mental health	Self-rated health	Mental health
Formal social support	−0.647*** (0.077)	−0.282*** (0.082)	−0.583* (0.259)	−0.417 (0.064)
Informal social support	−0.234** (0.156)	−0.231** (0.183)	−0.147 (1.036)	−1.410 (1.066)
Control variable	YES	YES	YES	YES

### 3.5. Endogenous elimination

**Fig 1:**
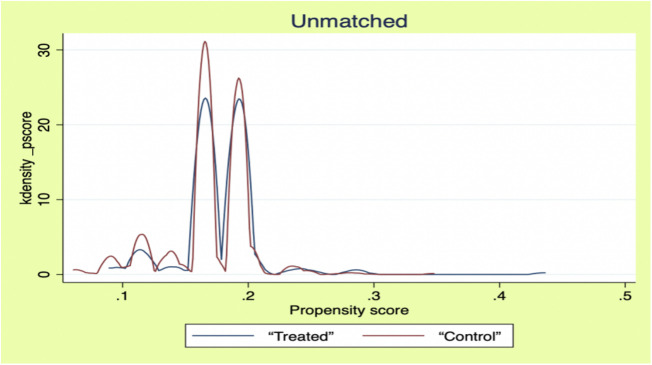
Unmatched kernel density function diagram.

**Fig 2:**
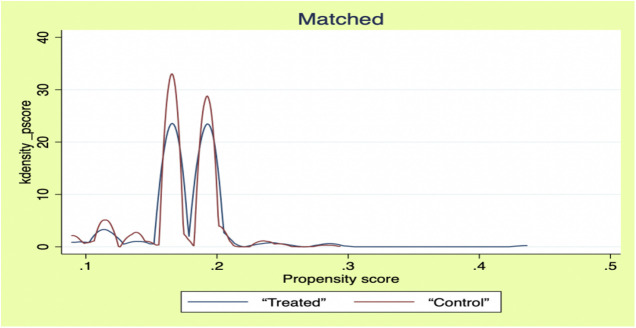
Matched kernel density function diagram.

The model may be endogenously biased due to self-selection bias in social support acceptance behavior due to differences in individual characteristics of male older adults, as well as the problem of bi-directional selection between health status and the need for social support (those in poorer health are more inclined to accept support). For this reason, this study used the propensity score matching (PSM) method to estimate the pure effect of social support on the health of older men. According to the PSM modeling requirements, the sample needs to pass the balance test before subsequent analysis. Table 5 shows that the samples were well-balanced after matching (standardized deviation <5%); the kernel density function plots (Figs 1 and 2) further confirmed that the sample distribution was discrete before matching, while the distribution patterns of the treatment and control groups converged after matching. This indicates that the PSM model effectively mitigates the selection bias problem and meets the analysis requirements.

**Table 5:** Sample balance test.

Variable	Matching condition	Mean		Bias(%)	Calibration deviation(%)	T-test	
		Treated	Control			t	P >|t|
Age	Unmatched	65.066	65.027	6.0	56.5	0.91	0.363
	Matched	65.027	65.010	2.6		0.64	0.524
Live place	Unmatched	1.070	1.141	−12.7	97.2	−1.69	0.092
	Matched	1.070	1.067	0.4		0.05	0.961
Education level	Unmatched	2.105	2.083	6.8	68.8	1.03	0.301
	Matched	2.102	2.094	2.1		0.23	0.820
Marital status	Unmatched	1.164	1.155	1.6	89.2	0.24	0.812
	Matched	1.165	1.165	−0.2		−0.02	0.984
Medical insurance	Unmatched	0.992	0.985	6.3	90.2	0.84	0.399
	Matched	0.992	0.991	0.6		0.08	0.935
Smoking	Unmatched	0.101	0.141	−12.0	79.9	−1.67	0.095
	Matched	0.102	0.094	2.4		0.30	0.766
Drinking	Unmatched	0.500	0.554	−10.9	95.2	−1.59	0.113
	Matched	0.498	0.495	0.5		0.06	0.953

In order to accurately estimate the pure effect of social support on the health of male older adults, three classical matching methods (K-nearest neighbor matching, intra-caliper K-nearest neighbor matching, and kernel matching) were used in this study [30]. The results in Table 6 show that in the self-rated health dimension, the pre-matching ATT was 0.583, and the three methods’ ATT values after matching were 0.588, 0.588, and 0.590, respectively; and in the mental health dimension the pre-matching ATT was 0.240, and the post-matching ATT values were 0.242, 0.246, and 0.242, respectively. all the matching methods showed an increase in the ATT values compared to the pre-matching values, which indicated that the Ignoring sample selectivity bias will lead to systematic underestimation of the health effect of social support. The PSM model effectively eliminated confounding errors and confirmed the underestimated improvement effect of social support on physical and mental health of male older adults.

**Table 6:** Matching estimation results of propensity score.

	Self-rated health				Mental health			
	Treated	Control	ATT	SE	Treated	Control	ATT	SE
Unmatched	3.195	2.612	0.583	0.067	1.855	1.615	0.240	0.006
Matched								
K-nearest neighbor matching	3.196	2.607	0.588	0.069	1.850	1.609	0.242	0.008
K-nearest neighbor calipers match	3.196	2.608	0.588	0.069	1.850	1.614	0.246	0.071
Kernel matching	3.196	2.605	0.590	0.068	0.850	1.608	0.242	0.070

## 4. Discussion

### 4.1. Summary of findings

The results of this study show that with the deepening of the aging degree, social support can significantly improve the health level of elderly men and has a positive effect on the improvement of their self-rated health and mental health. This result is basically consistent with the study of Wei [31]. The results of heterogeneity test showed that the effect of social support on the health of older men with lower education level is more obvious than that of older men with higher education level. The possible reason is that older people with higher education often have sufficient economic foundation and can use all resources to improve their health, so additional social support is not very necessary for them [32]. However, older men with less education often have less resources to improve their health, so they are very sensitive to and need additional social support, which will better improve their health [33]. The results of endogeneity test show that PSM model can eliminate the endogeneity problem of the model. If we do not eliminate the endogeneity of the model, on the one hand, we will underestimate the promoting effect of social support on self-rated health of elderly men; On the other hand, it also underestimated the effect of social support on improving mental health. In addition, this conclusion is still valid after the robustness test of the replacement econometric model.

### 4.2. Policy implications

At present, Chinese society has entered a deep aging wave, while the low birth rate and marriage rate are very obvious [34]. Relevant studies have shown that the elderly population of single men living alone in China is gradually increasing, and their health status is not optimistic [35–37]. Therefore, the health of the elderly men has become an important issue of academic and social concern. In this situation, it is of great practical significance to use social support to improve the health of elderly men. Current research on the relationship between social support and population health is well established and the findings of this paper are largely consistent with them [38,39]. However, in contrast to previous studies, this study highlights the impact of social support on the physical and mental health of older men as a group from a gender perspective, which contributes to our understanding of changes in the health of populations in the new developmental stage from a gender-differentiated perspective. In addition, by focusing on the relationship between social support and the physical and mental health of older men, the findings of this study are highly relevant to the United Nations Sustainable Development Goals (SDGs), particularly Goal 3 (Good Health and Well-Being) and Goal 10 (Reducing Inequality), and this study provides an empirical rationale and a concrete pathway for advancing the realization of these global goals. First, this study clearly shows that higher levels of social support significantly improve the mental health and physical health of older men, a finding that directly echoes the SDG 3 core requirement of “promoting well-being at all ages”. In the male population in particular, strengthening social support networks is important for preventing physical and mental health problems and reducing reliance on high-cost medical services, which is consistent with the preventive health care and health promotion strategies emphasized in SDG 3.

Second, our study focused specifically on the group of male older adults. Numerous studies have shown that old age itself may be a period of vulnerability due to factors such as physical decline, shifting social roles, and limited economic resources [40–41]. In turn, male older adults may face unique challenges, such as narrower social networks, limited emotional expression, and greater reluctance to seek help due to traditional gender roles, making them potentially disadvantaged or with specific needs in accessing and utilizing social support. The significant impact of social support on the mental and physical health of older men revealed in this study highlights the concern for possible risks of inequality in health and social participation for this group. Encouraging and facilitating the active participation of older men in social interaction and community integration is in itself a process of reducing social exclusion and improving intergenerational and intergroup understanding, which contributes to older men’s social participation and sense of belonging, which is a tangible manifestation of the realization of the “social, economic, and political inclusion” advocated by SDG 10 in the context of the older population.

According to the above research results, this paper puts forward the following policy recommendations: First, build a comprehensive social support system. The Chinese government should take the lead in building a comprehensive social support system, bring together communities, families, non-governmental organizations and other forces, strengthen the functions of community elderly care services, add dedicated service facilities, and provide health monitoring, rehabilitation guidance, and psychological counseling services. At the same time, the promotion of smart elderly care technology, the use of modern scientific and technological means to provide convenient health management and emergency rescue services for elderly men. In addition, social contact and emotional support for older men are enhanced through community activities and neighborhood assistance mechanisms. Second, strengthen the emotional bond between family and society. To enhance the emotional support of elderly men, families should improve the quality of care, accompany and communicate regularly, and pay attention to their psychological changes. At the same time, establish a neighborhood mutual assistance mechanism, encourage neighbors to help each other, and create a warm and harmonious community atmosphere. At the social level, it is necessary to strengthen publicity and advocacy, improve the attention of the whole society to the health problems of elderly men, and jointly create a social fashion of respecting the elderly, so that the elderly can feel the warmth and care from the family and society. Third, promote education and lifelong learning for the elderly. Relevant departments and social organizations should increase investment in education for the elderly, enrich educational resources, provide diversified learning courses and activities, especially for the interests and needs of elderly men, and set up practical learning courses. At the same time, a learning and exchange platform will be built to promote knowledge sharing and experience exchange among the elderly. The elderly are encouraged to actively participate in social activities for the public good, as far as their physical health allows, and to maintain their vitality and sense of self-worth through continuous learning and social participation, thereby promoting their physical and mental health.

### 4.3. Innovations and limitations

The marginal contributions of this paper are as follows: First, this paper further explores the impact of social support on the health of elderly men by using the newly published national data, which helps us to understand the changes in population health in the new development stage from the perspective of gender differences. Second, from the perspective of healthy aging for the first time, the effects of social support on elderly men at different ages and different levels of education were verified, providing theoretical reference for further understanding of the role of social support. Third, this paper measures social support from formal support and informal support, which enriches the connotation of social support. Fourthly, compared with previous studies, this paper estimates the pure effect of social support on the health of elderly men by using the PSM model, eliminating the endogeneity problem caused by sample selection bias. However, this paper also has some limitations: First, there may be omissions in the control variables selected in this paper. Although we have screened the control variables as comprehensively as possible, there are inevitably omissions. Secondly, due to the limitation of sample data, this paper measures social support from formal and informal support. However, there are many ways to measure social support. In the future, we will further study the impact of social support on the health of elderly men in different ways, to conduct a more comprehensive analysis. The above limitations are the direction that we will further deepen our research in the future.

## 5. Conclusions

In the context of increasing population aging and declining birth rate, it is of great practical significance to explore the impact of social support on the health of elderly men, which is of great practical significance to smoothly promote the strategy of healthy China and actively implement the strategy of population aging. Based on the newly published data of China Health and Retirement Longitudinal Study (CHARLS) in 2020, this paper analyzed the impact of social support on the health of elderly men by using Oder Probit, OLogit, PSM and other models. The results show that with the deepening of the aging degree, social support can significantly improve the health level of elderly men and has a positive effect on the improvement of their self-rated health and mental health. The results of heterogeneity test showed that the influence of social support on the health of elderly men had significant heterogeneity. The effect of social support on the health of older men with lower education level is more obvious than that of older men with higher education level. The results of endogeneity analysis show that PSM model can eliminate the endogeneity problem of the model. If we do not eliminate the endogeneity of the model, on the one hand, we underestimate the promoting effect of social support on self-rated health of elderly men; On the other hand, it also underestimated the effect of social support on improving mental health. In addition, the robustness test results show that this conclusion is still valid after the robustness test of the replacement econometric model.
